# Antibacterial Effects of Bulgarian Oregano and Thyme Essential Oils Alone and in Combination with Antibiotics Against *Klebsiella pneumoniae* and *Pseudomonas aeruginosa*

**DOI:** 10.3390/microorganisms13040843

**Published:** 2025-04-07

**Authors:** Silviya Mihaylova, Antoaneta Tsvetkova, Sylvia Stamova, Neli Ermenlieva, Gabriela Tsankova, Emiliya Georgieva, Katya Peycheva, Veselina Panayotova, Yulian Voynikov

**Affiliations:** 1 Medical College, Medical University of Varna, 9002 Varna, Bulgaria; antoaneta.tsvetkova@mu-varna.bg (A.T.); emiliya.georgieva@mu-varna.bg (E.G.); 2 Department of Pharmaceutical Chemistry, Faculty of Pharmacy, Medical University of Varna, 9002 Varna, Bulgaria; silvia.stamova@mu-varna.bg; 3 Department of Microbiology and Virology, Faculty of Medicine, Medical University of Varna, 9002 Varna, Bulgaria; neli.ermenlieva@mu-varna.bg (N.E.); gabriela.tsankova@mu-varna.bg (G.T.); 4 Department of Chemistry, Faculty of Pharmacy, Medical University of Varna, 9002 Varna, Bulgaria; peycheva@mu-varna.bg (K.P.); ivanova@mu-varna.bg (V.P.); 5 Department of Chemistry, Faculty of Pharmacy, Medical University of Sofia, 1000 Sofia, Bulgaria; y_voynikov@pharmfac.mu-sofia.bg

**Keywords:** antimicrobial resistance, essential oils, *Klebsiella pneumoniae*, *Pseudomonas aeruginosa*, carvacrol, thymol, Gram-negative bacteria, Bulgaria

## Abstract

Antimicrobial resistance (AMR) poses a global health threat, with multidrug-resistant (MDR) *Klebsiella pneumoniae* and *Pseudomonas aeruginosa* contributing to the burden, especially in Bulgaria. This study investigates recent resistance trends of these pathogens in Bulgaria and evaluates the antibacterial effects of commercially available oregano (*Origanum vulgare*) and thyme (*Thymus vulgaris*) essential oils (EOs), both alone and in combination with conventional antibiotics. The chemical composition of the EOs was analyzed using gas chromatography–mass spectrometry (GC-MS), revealing carvacrol (84.2%) as the main component in oregano EO and thymol (45.74%) in thyme EO. Minimum inhibitory concentration (MIC) and minimum bactericidal concentration (MBC) values of the EOs were determined using the serial dilution method. Oregano EO exhibited lower MIC and MBC values (0.039–1.250%) compared to thyme EO (0.156–5.000%) against both pathogens. The disk diffusion assay showed that oregano EO exhibits more potent antibacterial effects than thyme EO in *Klebsiella pneumoniae* and *Pseudomonas aeruginosa* strains. The findings highlight the potential of EOs as adjunctive therapies to enhance the efficacy of conventional antibiotics against MDR *Klebsiella pneumoniae* and *Pseudomonas aeruginosa* strains in Bulgaria.

## 1. Introduction

Antimicrobial resistance (AMR) has emerged as one of the most significant global health threats of the 21st century, imposing substantial morbidity, mortality, and a high economic burden [1]. The World Health Organization (WHO) estimates that AMR could result in additional healthcare costs of USD 1 trillion by 2050, with global gross domestic product (GDP) losses ranging from USD 1 trillion to USD 3.4 trillion per year by 2030 [2]. This burden is especially severe in low- and middle-income countries, where limited resources exacerbate the impact of multidrug-resistant (MDR) infections. Notably, the development of novel antibiotics alone has proven insufficient to curb AMR, given the complexities and high costs associated with drug discovery and development [3]. Indeed, over the past two decades, no new class of antibiotics has been successfully introduced, underscoring the urgent need for innovative strategies [1].

Among the pathogens contributing most significantly to AMR, *Pseudomonas aeruginosa* (*P. aeruginosa*) and *Klebsiella pneumoniae* (*K. pneumoniae*) demonstrate high adaptability and resistance profiles. Both organisms have developed mechanisms such as extended-spectrum β-lactamases (ESBLs) and carbapenemases, efflux pumps, porin modifications, and horizontal gene transfer of resistance elements [4]. These capabilities render them difficult to treat with conventional antibiotics, leading the WHO to classify them among the highest-priority pathogens [5,6]. A systematic analysis from the Global Antibiotic Research & Development Partnership (2024) [1] showed that mortality attributed to carbapenem-resistant Gram-negative organisms increased by over 89,000 deaths between 1990 and 2021.

*P. aeruginosa* presents a serious therapeutic challenge for the treatment of both community-acquired and nosocomial infections, and selection of the appropriate antibiotic to initiate therapy is essential to optimizing clinical outcome [7]. *P. aeruginosa* is one of the most biochemically adaptable bacterial species, often found in hospital-acquired infections, especially in long-term hospitalized patients on antibiotic therapy, and also in sewage. This pathogen ranks among the four most common pathogens in European hospitals and is associated with various ICU infections (uroinfections, pneumonias, and blood infections). *P. aeruginosa* is known for its ability to form biofilms, which are responsible for approximately 65% of nosocomial infections [8]. In addition to the severe course of pseudomonad infections, the etiological treatment of these infections is extremely difficult due to the innate and acquired antibiotic resistance. *P. aeruginosa* not only emerged as a multidrug-resistant (MDR) pathogen, but also evolved as extensively drug-resistant (XDR) and pandrug-resistant (PDR) strains as well [9]. The results reported by EARS Net for 2020 place Bulgaria in the first position in terms of the share of ceftazidime (54.3%) and fluoroquinolone-resistant (52.9%) invasive *P. aeruginosa*. In 2023, the percentage of isolates with a resistance phenotype decreased to 39.3 and 31.0, respectively, but remained two times higher than the EU average [10].

*K. pneumoniae* is a non-motile Gram-negative opportunistic pathogen that belongs to the Enterobacteriaceae family. The genera within the Enterobacteriaceae family are significant contributors to three of the four main categories of healthcare-associated infections (HAI) identified by the Centers for Disease Control and Prevention (CDC): central line-associated bloodstream infections (CLABSI), catheter-associated urinary tract infections (CAUTI), and surgical site infections (SSI) [11]. *K. pneumoniae* accounts for approximately 10% of nosocomial bacterial infections [12]. A comprehensive study from 2022 examined the burden of bacterial antimicrobial resistance in the WHO European region during 2019 [13]. The highest crude mortality rates for *K. pneumoniae* resistant to any antibiotic were observed in Bulgaria, with a mortality rate per 100,000 population of 4.59 [13].

The capacity of both species to form biofilms, which enhance tolerance to antimicrobials and immune responses, remains a key virulence factor [14]. These complexities have prompted the WHO to update its 2024 Bacterial Priority Pathogens List (BPPL), placing carbapenem-resistant *K. pneumoniae* (CRKP) at the top of the *critical group*, followed by third-generation cephalosporin-resistant *K. pneumoniae*, while carbapenem-resistant *P. aeruginosa* (CRPA) retains a *high priority* ranking [2]. A systematic analysis of the Global Antibiotic Research & Development Partnership, published in 2024, showed that annual mortality caused by carbapenem-resistant Gram-negative organisms increased by 89,200 deaths (50,900 to 127,000) between 1990 and 2021, more than any antibiotic class over that period [1].

There was a significant increase in infections caused by these pathogens during the COVID-19 pandemic, with *K. pneumoniae* and *P. aeruginosa* being among the most frequently isolated bacteria in hospital settings. This rise is attributed to factors such as increased hospital admissions, prolonged stays, and the extensive use of broad-spectrum antibiotics during the pandemic [15].

In line with these findings, data from the European Centre for Disease Prevention and Control (ECDC) [16] indicate a higher prevalence of carbapenem-resistant *K. pneumoniae* (CRKP) in Eastern European countries. For instance, in 2021, Greece reported a CRKP prevalence of 73.7%, while Romania, Serbia, and Bulgaria reported rates of 54.5%, 62.7%, and 46.3%, respectively. In contrast, Germany and France both reported a rate of 0.8%.

These trends highlight the importance of exploring not only new antibiotics, but also alternative therapeutic strategies that might enhance the efficacy of existing treatments. Essential oils have been studied for their antimicrobial properties and potential to combat resistant bacterial strains. Essential oils, particularly those rich in phenolic monoterpenes, such as carvacrol and thymol, have demonstrated antibacterial activity, including synergy with conventional antibiotics, thereby offering promising complementary or adjunctive therapies [4,17,18,19].

Oregano (*Origanum vulgare*) and thyme (*Thymus vulgaris*) essential oils (EOs) are well-known for their broad-spectrum antimicrobial properties. Numerous studies have shown that both oils exert significant inhibitory effects against a range of bacteria, including common foodborne and clinical pathogens [20,21].

Both oregano and thyme EOs share similar mechanisms of action owing to their chemical makeup. Their hydrophobic components enable the oils to partition into bacterial cell membranes and disrupt membrane integrity [22]. Key constituents such as carvacrol (abundant in oregano oil) and thymol (dominant in thyme oil) insert into the lipid bilayer, causing the increased permeability of the cell membrane and losses of membrane potential. This leads to the leakage of vital cell contents and ions, ultimately resulting in cell death. For example, Xu et al. (2008) [23] demonstrated that carvacrol and thymol at ~0.02% (200 mg/L) caused membrane permeabilization and depolarization in *Escherichia coli*, explaining their bactericidal effect. Other studies show that these phenolics can act as proton exchangers, collapsing the proton motive force and ATP synthesis in bacteria [24]. In addition to membrane damage, oregano and thyme EOs may interfere with bacterial enzymes and quorum sensing pathways, and they have been shown to inhibit biofilm formation in several species [24]. This multi-targeted mode of action is significant because it reduces the likelihood that bacteria can develop resistance—indeed, no resistance development was observed in bacteria after repeated exposure to sublethal doses of oregano oil in one study [24].

An essential oil is defined as a steam-distilled extract of twigs, leaves, woods, seeds, exudates, fruits, flowers, barks, and roots. The International Fragrance Association (IFRA) [25] lists 237 oils that meet this definition and are widely used in oral antiseptic solutions, food preservation, additives and flavors, perfumes, cosmetics industries, toothpastes, cleaning agents, and air fresheners. Currently, the European Medicines Agency [26] has approved 16 essential oils (aetheroleum) as traditional herbal medicinal products, as follows: Peppermint oil (*Mentha piperita* L.), Lavender oil (*Lavandula angustifolia Mill.*), Tea tree oil (*Melaleuca alternifolia* (*Maiden & Betche*) *Cheel*), Eucalyptus oil (*Eucalyptus globulus Labill.*), Rosemary oil (*Rosmarinus officinalis* L.), Lemon oil (*Citrus limon* (L.) *Osbeck*), Chamomile oil (*Matricaria chamomilla* L.), Anise oil (*Pimpinella anisum* L.), Fennel oil (*Foeniculum vulgare Mill.*), Caraway oil (*Carum carvi* L.), Cornmint oil (*Mentha arvensis* L.), Sage oil (*Salvia officinalis* L.), Thyme oil (*Thymus vulgaris* L.), Juniper oil (*Juniperus communis* L.), Pine oil (*Pinus sylvestris* L.), and Lemon balm oil (*Melissa officinalis* L.).

The chemical characterization of many essential oils reveals the presence of only 2–3 major components at a fairly high concentration (20–70%) compared to other components present in trace amounts [27]. Most essential oils are composed of terpenes, terpenoids, and other aromatic and aliphatic constituents with low molecular weights. Terpenes or terpenoids are synthesized within the cytoplasm of the cell through the mevalonic acid pathway. Some of the major compounds of oregano and thyme essential oils include monoterpene hydrocarbons (p-cymene, γ-terpinene), sesquiterpene hydrocarbons (β-caryophyllene), monoterpene alcohols (linalool) and monoterpene phenols (thymol, carvacrol).

The use of EOs often results in various effects that can disrupt the integrity of the phospholipid bilayer, compromise the functionality and composition of the plasma membrane, cause the depletion of crucial intracellular components, and inhibit the activity of hydrolytic enzymes [28]. In addition, EOs have several ways of affecting microbial biofilm formation, such as causing cell membrane rupture, blocking quorum sensing (QS) activity, inhibiting bacterial motility, preventing cell adhesion, reducing extracellular polymeric substances formation, and suppressing cell proliferation [29]. Notably, several studies describe the synergistic or complementary actions of essential oils with conventional antibiotics against resistant strains [30,31,32]. The inhibition of QS may reduce pathogenicity, antibiotic resistance, and biofilm formation in systemic and local infections. Szabó et al. (2010) claim that essential oils of rose, geranium, lavender, and rosemary were the most potent QS inhibitors. Eucalyptus and citrus oils showed a moderate effect, while chamomile, orange, and juniper oils were ineffective [33]. In a review article, Shariati et al. indicated that the components in essential oils, such as curcumin, carvacrol, thymol, eugenol, cinnamaldehyde, terpinene-4-ol, linalool, pinene, linoleic acid, and geraniol, are the major natural elements most extensively utilized for the management of the *P. aeruginosa* biofilm community [29]. The findings of Walczak et al. indicate that carvacrol inhibited biofilm formation by up to 74–88% for *P. aeruginosa* and decreased the enzyme activity of the biofilm by up to 40–100% [34].

This study investigates recent resistance trends in two clinically significant Gram-negative pathogens—*K. pneumoniae* and *P. aeruginosa* in Bulgaria. Given the persistently high rates of antimicrobial resistance (AMR) in the region, we first analyze resistance patterns to established antibiotic classes. Recognizing the growing need for adjunctive therapies against multidrug-resistant (MDR) strains, we then evaluate the antibacterial effects of two commercially available essential oils from the *Lamiaceae family* (oregano and thyme). As part of this investigation, we also analyze these oils’ chemical compositions to identify their principal bioactive constituents, and we test their efficacy against *K. pneumoniae* and *P. aeruginosa* isolates from clinical wound and sputum samples.

## 2. Materials and Methods

### 2.1. Essential Oils

Essential oils (*O. vulgare* and *T. vulgaris*) were purchased from a commercial market and stored in dark glass bottles at a temperature of 4 °C without access to light throughout the duration of the experiments. According to the manufacturer, the essential oils were certified as 100% pure, with organically sourced ingredients. The specific chemotype or subspecies of each essential oil was not reported. The organic solvent (DMSO) used in this study was obtained from Merck (Darmstadt, Germany).

### 2.2. Microorganisms

A total of 7 bacterial strains, comprising 5 clinical isolates and 2 reference strains, were included in this study. Reference strains of *Pseudomonas aeruginosa ATCC 27853* and *Klebsiella pneumoniae ATCC 13883* were obtained from Ridacom, Bulgaria, under the MicroSwap trademark. Clinical isolates were collected, identified and stored at −18 °C in glycerol medium in a microorganism bank at the Varna Medical College. The use of clinical strains received approval from the Commission of Ethics in Scientific Research, Medical University of Varna, Bulgaria (Protocol № 116, 28 April 2022). For the purposes of the research in this work, they were cultured initially in brain heart infusion broth for 24 h and then on blood agar for another 24 h.

### 2.3. GC-MS Analysis

An Agilent Technologies 7890A gas chromatograph (Santa Clara, CA, USA) equipped with a flame ionization detector (FID) and a 5975C mass selective detector (MSD) (Santa Clara, CA, USA) was used. The GC was fitted with a Stabilwax (Restek, Bellefonte, PA, USA) capillary column (30 m × 0.25 mm ID × 0.25 µm film thickness). The oven program was initiated at 65 °C, then ramped to 170 °C at a rate of 1.5 °C/min, with a total run time of 70 min. Both the injector and detector temperatures were maintained at 250 °C. Hydrogen and helium were used as carrier gases at a flow rate of 0.8 mL/min. The MSD scanned a mass range of *m*/*z* 40–450. Samples (1.0 µL) were injected in split mode (100:1). Compound identification was performed by comparing retention times and relative Kovats retention indices (RI) with those of authentic standards and by comparing mass spectral data with the NIST’08 (National Institute of Standards and Technology, Gaithersburg, MD, USA) and Adams library [35].

### 2.4. Determination of the Minimum Inhibitory Concentration (MIC) and Minimum Bactericidal Concentration (MBC)

The double-dilution method recommended by EUCAST (European Committee on Antimicrobial Susceptibility Testing) [36] was used to determine the MIC of essential oils (thyme and oregano) using Mueller–Hinton liquid medium (MHB) (Merck, Darmstadt, Germany) as the testing medium. Initially, a freshly cultured microbial inoculum of the studied bacterial strains was prepared using MHB and standardized to the 0.5 McFarland standard (density of 106 CFU/mL).

Essential oils were dissolved in 1% dimethyl sulfoxide (DMSO) to create a stock solution at 10% (*v*/*v*), and serial two-fold dilutions were prepared, ranging from 0.039% (*v*/*v*) to 5% (*v*/*v*), with a final volume adjusted to 1 mL. Different concentrations of EOs (100 μL) were added to the sterile tubes, after which 100 μL of the bacterial suspension was added, resulting in a final volume of 200 μL. For the assessment of bacterial growth, both positive controls (comprising MHB and microbial suspension) and negative controls (without inoculum) were integrated into the experimental setup. The samples were incubated at 37 °C for a duration of 24 h. The MIC was determined to be the lowest concentration of the EO that inhibited visible growth of the microorganisms. To determine the MBC values, 10 μL samples from clear wells of the MIC tests were subcultured on Mueller–Hinton agar plates. The MBC was classified as the lowest concentration of EO that resulted in a 99.9% reduction in the initial inoculum. All experiments were performed in triplicate, the MICs and MBCs were recorded as concentrations (*v*/*v*), and the results are expressed as the means of three determinations. The study has revealed the antibacterial properties of the essential oils against the tested strains.

### 2.5. Disk-Difusion Assay

The in vitro determination of the susceptibility patterns of the clinical isolates and the antimicrobial activity of antibiotic combinations with EOs was conducted using the Kirby–Bauer disk diffusion test [37]. Antibiotic disks—gentamycin (GEN, 10 µg), ciprofloxacin (CIP, 5 µg), ceftriaxone (CTR, 30 μg), and ceftazidime (CAZ, 10 μg) (HiMedia Laboratories GmbH, Einhausen, Germany)—were provided by Ridacom, Sofia, Bulgaria. Bacterial inocula were adjusted to an optical density equivalent to 0.5 McFarland standard and streaked onto Mueller–Hinton agar plates (HiMedia, provided by Ridacom, Bulgaria) using a sterile swab. The petri dishes were incubated for 24 h at 37 °C. Subsequently, in each medium with the culture of the corresponding microbe, two disks (d = 6 mm) were placed in the following combinations and concentrations of active agents: (1) factory-prepared antibiotic disk in a concentration set according to EUCAST, 2025 standards, additionally impregnated with 20 μL of 0.1% (*v*/*v*) thyme EO; (2) factory-prepared antibiotic disk in a concentration set according to EUCAST, 2025 standards, additionally impregnated with 20 μL of 0.1% (*v*/*v*) oregano EO. The selection of antibiotics against each of the test strains was made according to the EUCAST, 2025 instructions [38]. Disks impregnated in 1% (*v*/*v*) DMSO were used as a negative control, while 6 mm factory-prepared disks of the antibiotics gentamycin (GEN, 10 µg), ciprofloxacin (CIP, 5 µg), ceftriaxone (CTR, 30 μg), and ceftazidime (CAZ, 10 μg) were used as the positive controls. Each experiment was carried out in triplicate, the diameters of the inhibition zone were recorded in millimeters (mm), and the results are expressed as the mean of three determinations ± the standard deviation (SD). The inhibition zones were measured in terms of diameter (mm) and interpreted for their effects on the respective microbe (S—sensitive or R—resistant), following the EUCAST, 2025 instructions [38].

### 2.6. Assessment of the FIC Index

To determine the interactions between ciprofloxacin and thyme EO, a checkerboard microtiter assay was performed in a 96-well microplate. Briefly, 1:1 volumes (50 µL; 50 µL) of the tested substances were added to microtitre plates with 0.1 mL of medium. The dilutions were prepared beforehand as described in the MIC methodology. Serial dilutions of two antimicrobial agents were mixed together so that each row and column contained a fixed amount of the first agent and increasing amounts of the second one. Ten serial two-fold dilutions of ciprofloxacin (0.125 μg to 64 μg) and eight serial two-fold dilutions of thyme EO (0.039% to 5% (*v*/*v*)) were prepared. Subsequently, 0.1 mL of fresh bacterial suspension (0.5 MF) was added to each well and the microtitre plates were incubated under optimal conditions (37 °C for 24 h). To assess the interactions, the data obtained were analyzed using the fractional inhibitory concentration index (FICI) as follows equation: A/MICa + B/MICb = FICA + FICB = FICI. Here, A and B are the MICs of each antimicrobial agent in combination (in a single well), and MICa and MICb are the MICs of each drug individually.

## 3. Results and Discussion

### 3.1. Public Health Implications

**Figure 1:**
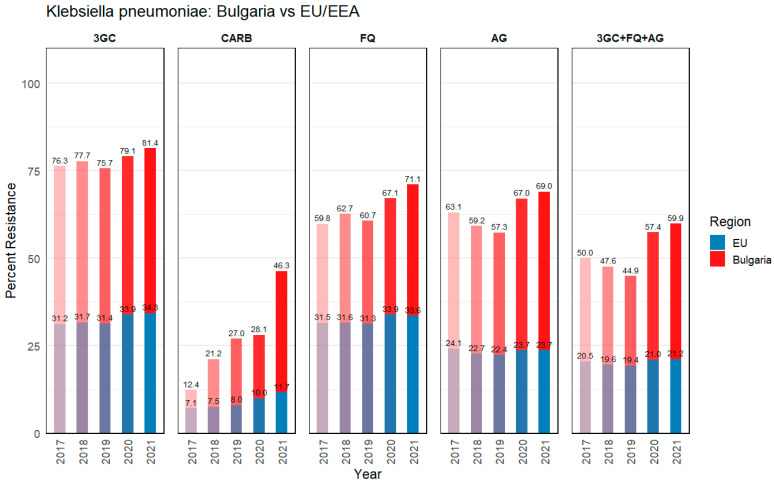
*Klebsiella pneumoniae* (percent resistance) data for Bulgaria and the EU/EEA. The bars (red for Bulgaria, blue for EU/EEA) indicate the percentages of isolates resistant to 3GC, CARB, FQ, AG and 3GC + FQ + AG. Data were extracted from the European Centre for Disease Prevention and Control, Antimicrobial resistance surveillance in Europe 2023, data from 2017 to 2021 [16]. EU/EEA population-weighted mean values are presented, number of EU/EEA countries: 30 in 2017–2019, 29 in 2020–2021 (Excluding the UK). Abbreviations: 3GC, third-generation cephalosporins; CARB, carbapenems (imipenem/meropenem); FQ, fluoroquinolones (cipro-/levo-/ofloxacin); AG, aminoglycosides (gentamicin/netilmicin/tobramycin); 3GC + FQ + AG, combined resistance to third-generation cephalosporins, fluoroquinolones, and aminoglycosides.

**Figure 2:**
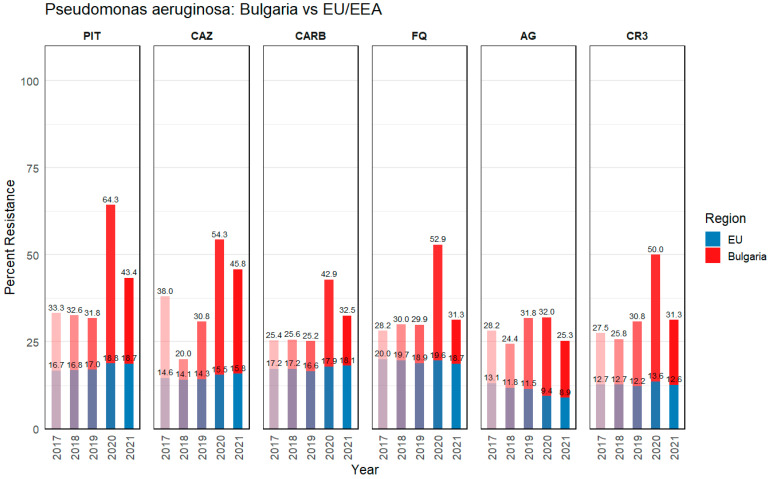
*Pseudomonas aeruginosa* (percent resistance) data for Bulgaria and the EU/EEA. Bars (red for Bulgaria, blue for EU/EEA) indicate the percentage of isolates resistant to PIT, CAZ, CARB, FQ, AG and CR3. Data were extracted from the European Centre for Disease Prevention and Control, Antimicrobial resistance surveillance in Europe 2023, data for 2017 to 2021 [16]. EU/EEA population-weighted mean values are presented, number of EU/EEA countries: 30 in 2017–2019, 29 in 2020–2021 (Excluding the UK). Abbreviations: PIT, piperacillin–tazobactam; CAZ, ceftazidime; CARB, carbapenems; FQ, fluoroquinolones; AG, aminoglycosides; CR3, combined resistance to three or more antibiotic groups (among PIT, CAZ, CARB, FQ, and AG).

The data presented in Figure 1 and Figure 2 indicate the percentage of isolates with resistance phenotypes (3GC, CARB, FQ, AG, 3GC + FQ + AG) out of the total number of reported invasive isolates. The total number includes isolates reported to EARS-Net, which collects antimicrobial resistance data from invasive isolates regardless of the origin of infection. Therefore, data may include both nosocomial and community infections. According to the Antimicrobial resistance surveillance report in Europe data, for 2017 to 2021 [16], Bulgaria maintained worryingly high rates of combined antibiotic resistance in isolates (blood or CSF samples) from both *K. pneumoniae* and *P. aeruginosa*, as shown in Figure 1 and Figure 2. These bar charts illustrate the percentages of invasive *K. pneumoniae* (Figure 1) and *P. aeruginosa* (Figure 2) isolates identified as resistant to various antibiotics or antibiotic combinations in Bulgaria (red bars) in contrast to the population-weighted mean across the EU/EEA (blue bars).

#### 3.1.1. *Klebsiella pneumoniae*

The observed phenotypic resistance in all tested antibiotic groups for *K. pneumoniae* in Bulgaria was significantly higher compared to the EU/EEA levels (Figure 1). In Bulgaria, *K. pneumoniae* consistently exhibited elevated resistance to third-generation cephalosporins (3GC), fluoroquinolones (FQ), aminoglycosides (AG), and carbapenems (CARB) compared to the EU/EEA means, demonstrating a serious threat to effective antimicrobial therapy. For example, resistance to third-generation cephalosporins (3GC) in Bulgaria averaged 78%, whereas the overall EU/EEA was around 33%. Carbapenem-resistant *K. pneumoniae* strains have posed a critical challenge in Bulgarian healthcare settings, with an observed average (years 2017–2021) resistance of 27%, compared to 9% in the EU/EEA, indicating a more extensive reliance on last-line agents and a likely higher local prevalence of carbapenemase-producing strains. Between 2017 and 2021, *K. pneumoniae* in the EU/EEA showed a marked and steadily increasing trend of carbapenem resistance, reaching 11.7% by 2021, with further evidence of combined resistance to multiple antimicrobial groups in a substantial proportion of isolates. Fluoroquinolone (FQ) resistance in Bulgaria’s *K. pneumoniae* was also roughly double that of the EU/EEA average (64% vs. 32%), and a parallel trend emerged for combined resistance to multiple antibiotic classes. The same goes for aminoglycosides’ (AG) resistance, where, since 2019, there had been increases in resistance isolates, generally reaching three-fold higher in Bulgaria compared to EU/EEA (63% vs. 23%). Additionally, for the 5-year period (2017–2021), more than half (52%) of the isolates in Bulgaria show multidrug resistance (MDR) to three or more antibiotic classes (3GC + FQ + AG), a stark contrast to the EU/EEA average of 20%. These high rates suggest potential gaps in infection prevention measures and antimicrobial stewardship programs, both of which might warrant reinforcement at the national level. The combined resistance (3GC + FQ + AG) does show a similar trend.

#### 3.1.2. *Pseudomonas aeruginosa*

Similarly, *P. aeruginosa* exhibited significantly higher resistance rates in Bulgaria compared to the EU/EEA (Figure 2). Looking at the average values (years 2017–2021), antimicrobial resistance (AMR) to piperacillin–tazobactam (41% BG vs. 17% EU/EEA), ceftazidime (38% BG vs. 15% EU/EEA), and carbapenems (30% BG vs. 17% EU/EEA) demonstrates a concerning trend. In general, in the year 2020, there was a marked increase in all presented antibiotic resistances. Piperacillin–tazobactam (PIT) resistance, for instance, had a mean of roughly 41% in Bulgaria versus 17% EU/EEA-wide, with especially large year-to-year fluctuations. Carbapenem (CARB) resistance followed a similar pattern of elevated rates, but is somewhat less extreme than with *K. pneumoniae*. Notably, Bulgaria’s aminoglycoside (AG) resistance for *P. aeruginosa* averages about 28%, in contrast to an EU/EEA figure near 11%. Local prescribing habits and healthcare-associated factors may have led to a more entrenched multidrug-resistance profile for *P. aeruginosa*. Carbapenem-resistant *P. aeruginosa* strains have particularly limited treatment options, often necessitating the use of combination therapies or colistin, which itself has toxicity concerns associated with it. The high levels of carbapenem resistance (30%) in Bulgaria are alarming, as carbapenems are a last-line treatment for severe *P. aeruginosa* infections. Moreover, 35% of isolates in Bulgaria exhibit fluoroquinolone (FQ) resistance, which is considerably higher than the EU/EEA average of 19%. The overall prevalence of multidrug resistance (CR3) among *P. aeruginosa* in Bulgaria (33%) is also worrying, as it limits the availability of therapeutic strategies.

In general, *P. aeruginosa* demonstrated comparatively lower overall resistance percentages than *K. pneumoniae* [16]. Notably, both pathogens reflected a north-to-south and west-to-east gradient across European countries, with higher resistance rates observed in southern and eastern regions. These observations highlight the continuing challenge of effectively controlling infections caused by these two species, with the pandemic warranting the need for enhanced surveillance, infection prevention and control measures to curb the progression of resistance in these pathogens.

The data presented in Figure 1 and Figure 2 show that Bulgaria has consistently reported higher levels of resistance for both *K. pneumoniae* and *P. aeruginosa* across all critical antibiotic classes. The results show that the effectiveness of available therapeutic modalities is decreasing, making it more difficult to treat infections; prolonging hospital stays; increasing the financial burden on healthcare facilities and patients; and increasing mortality rates. These findings highlight the urgent need for enhanced antimicrobial treatment programs, infection control measures, and surveillance strategies to curb the rapid spread of MDR pathogens in Bulgarian healthcare.

Overall, Bulgaria’s elevated resistance rates underscore the importance of focused surveillance, prudent antibiotic administration, and consistent infection-control strategies in both hospital and community settings. The contrast with EU/EEA averages suggests there is room for knowledge exchange on best practices, particularly regarding the early detection of resistant strains and optimized antimicrobial regimens that can lower the pressure selecting for highly resistant organisms.

### 3.2. Antibiotic Sensitivity Profile of Clinical Isolates

The sensitivity of clinical isolates to antibiotics is presented in Table 1. Disk diffusion susceptibility testing revealed that the *K. pneumoniae* isolates showed different sensitive responses to antibiotics tested. Clinical isolate KP17 (from sputum) was resistant to ceftriaxone (CTR), while clinical isolate KP18 (from wound) was resistant to ciprofloxacin (CIP) and intermediate to third-generation cephalosporin (both CTR and CAZ). Data for *P. aerugonisa* isolates show that they were intermediate to ciprofloxacin (CIP) and ceftazidim (CAZ), with the exception of PA2, which is sensitive to CAZ.

The presence of intermediate susceptibility (I) in both *K. pneumoniae* and *P. aeruginosa* requires strict monitoring and precise molecular diagnostics, and often leads to therapeutic failures. Although only two clinical isolates of *K. pneumoniae* were included in the study, the data confirm the trend of a serious antimicrobial resistance problem in Bulgaria. As shown in Figure 2, the average percentages of the phenotypic resistance of *K. pneumoniae* to third-generation cephalosporins and fluoroquinolones for Bulgaria are 78% and 64%, respectively.

### 3.3. Antibacterial Activity of Oregano and Thyme Essential Oils Alone and in Combination with Conventional Antibiotics Against K. pneumoniae and P. aeruginosa Strains

This section presents the results of antibacterial activity studies of oregano and thyme EOs alone and in combination with conventional antibiotics against *K. pneumoniae* and *P. aeruginosa* strains. Table 2 shows the Minimum Inhibitory Concentration (MIC) and Minimum Bactericidal Concentration (MBC) values for oregano and thyme essential oils against different strains of *K. pneumoniae* and *P. aeruginosa* alone, while Table 3 and Table 4 present the diameters of inhibition zones of conventional antibiotics alone and in combination with 0.1% oregano EO and 0.1% thyme EO against *K. pneumoniae* strains and *P. aeruginosa* strains, respectively.

The results in Table 2 indicate that oregano and thyme EOs exhibited inhibitory and bactericidal activity against *K. pneumoniae* and *P. aeruginosa*. The lowest MIC value (0.039%) was observed for oregano EO against *K. pneumoniae* (KP17 and KP18), while the highest MIC value (5.000%) was found for thyme EO against *P. aeruginosa* (PA12). KP17 and KP18 (clinical isolates) showed markedly lower oregano EO MIC (0.039%) and MBC (0.039%) compared to *K. pneumoniae ATCC13883*, suggesting these clinical isolates are more susceptible to oregano EO. While oregano EO is very potent (as low as 0.039%) against certain clinical isolates, thyme EO remains effective, but at generally higher concentrations (0.156–0.312%). Among all *P. aeruginosa* strains, PA13 was notably more sensitive to oregano EO, while PA2 and PA12 needed higher doses. In general, oregano EO showed stronger activity at lower concentrations than thyme EO across most strains, especially with KP17, KP18, and PA13, which appear highly sensitive to oregano.

These findings align with those of a previous study by Ben Selma et al. [4], which reported the significant antibacterial activity of *Thymus algeriensis* EO against multidrug-resistant (MDR) *K. pneumoniae* and *P. aeruginosa*, with MIC values as low as 0.156 mg/mL. Similarly, Veljovic et al. reported that oregano EO rich in carvacrol and thymol exhibited strong antibacterial activity against respiratory pathogens [39]. Also, carvacrol and thymol, the primary components of oregano and thyme EOs, have been previously recognized for their potent antibacterial properties. According to da Silva et al. (2023), carvacrol exhibited MIC values of 81–161 µg/mL against *P. aeruginosa* [40]. Furthermore, Dong et al. demonstrated that synthetic derivatives of carvacrol and thymol exhibited enhanced antibacterial potency [5].

#### 3.3.1. Monitoring the Sensitivity of Bacterial Strains When Combining Essential Oils with Antibiotic

Table 3 shows the antibiotic susceptibility patterns of *K. pneumoniae* ATCC13883 (reference) and two clinical isolates (KP17, KP18) when four antibiotics (ceftriaxone [CTR], gentamicin [GEN], ciprofloxacin [CIP], ceftazidime [CAZ]) are used alone or combined with 0.1% (*v*/*v*) oregano EO or thyme EO. The inhibition zone diameter (mm) and susceptibility categories (S, R) are presented.

*K. pneumoniae* ATCC13883 was sensitive (S) to all tested antibiotics alone. However, the addition of 0.1% oregano or thyme EO in some cases increased the zone slightly (e.g., GEN alone = 20 mm vs. GEN + oregano EO = 30 mm, or GEN + thyme EO = 25 mm). This suggests that even when a strain is already sensitive, adding EOs may amplify antibiotic performance. CIP alone yielded a 43 mm zone, which remained nearly the same with EOs (44–45 mm). The same can be deduced for cephalosporins (CTR and CAZ), as the addition of EOs to cephalosporins did not notably alter the bacterial susceptibility. KP17 presented initially as resistant (R) to CTR alone (22 mm), shifting to sensitive (S) with oregano (30 mm) or intermediate (I) with thyme (26 mm) EO. For CIP, the zone diameter was 28–31 mm in all conditions, consistently indicating sensitivity, yet a small enhancement was observed (e.g., 29 mm alone vs. 31 mm with oregano EO). The zone for GEN alone (22 mm) remained in the sensitive range, and co-incubation with oregano or thyme EO (21–22 mm) did not drastically change the zone size or classification. Similarly, CAZ remained in the sensitive category (22 mm alone vs. up to 24 mm with oregano EO). KP18 appeared to be intermediate to CTR alone (24 mm) and remained intermediate (24 mm) with both EOs. Thus, unlike KP17, adding 0.1% EO did not convert CTR from intermediate to sensitive. For GEN, all tested conditions fell into the sensitive range (18–22 mm). Meanwhile, CIP alone yielded a smaller zone (19 mm) categorized as resistant (R). Even with oregano or thyme EO, the CIP zone (15–24 mm) fluctuated between R and I. Lastly, CAZ alone (20 mm) and its combinations remained in the intermediate category for KP18.

The results reveal that the addition of oregano EO with ceftriaxone leads to a change in KP17 sensitivity (shifting R to S), while combination CIP with thyme EO produced an inhibition zone smaller (15 mm) than CIP alone (19 mm) in KP18.

Table 4 focuses on CIP and CAZ against four *P. aeruginosa* strains (ATCC27853, PA2, PA12, PA13). Each strain is tested with antibiotic alone or with 0.1% oregano or thyme EO. The inhibition zone diameter (mm) and susceptibility categories (S, R) are presented. *P. aeruginosa* ATCC27853 was sensitive (S) to CIP (51 mm), and adding oregano EO or thyme EO did not notably alter the effect on the zone (52 mm). CAZ alone produced a large zone (55 mm), indicating sensitivity (S). Susceptibility to CAZ was slightly enhanced by both oregano EO (58 mm) and thyme EO (54 mm). PA2 also showed intermediate susceptibility to CIP alone (36 mm) and with thyme EO (41 mm). With added oregano EO, the sensitivity was increased (55 mm). For CAZ, PA2 was sensitive at 50 mm, and combining with oregano EO raised it to 54 mm (still S), whereas with added thyme EO, the susceptibility did not show improvement. Thus, oregano EO showed an improvement with CIP and CAZ, while thyme EO did not. The clinical isolate PA12 was intermediate to CAZ (22–23 mm) under all conditions, and the addition of thyme or oregano EO (22–23 mm) did not change the classification. Furthermore, an intermediate susceptibility of PA12 to CIP alone and with thyme EO (39 mm) was observed. Only the addition of oregano EO was observed to change susceptibility, with an inhibition zone of 51 mm. PA13 showed intermediate sensitivity (40 mm) to CIP alone, which remained the same (39–41 mm) with both EOs. Similarly, CAZ alone at 21 mm was intermediate, as was 23 mm with oregano EO. In general, the data suggest that for *P. aeruginosa*, adding 0.1% oregano EO could boost the CIP zone in tested strains ATCC27853, PA2 and PA12 (from I to S). Thyme EO tended to have smaller or negligible effects.

Taken collectively, the data show that oregano EO consistently exhibits more potent antibacterial effects than thyme EO across *K. pneumoniae* and *P. aeruginosa* strains, as shown both in direct MIC/MBC measurements and in combined antibiotic disk diffusion assays. While increasing antimicrobial activity is demonstrated in some combinations (e.g., ceftriaxone + oregano EO for KP17, ciprofloxacin + oregano EO for certain *P. aeruginosa* isolates), the magnitude of effect depends heavily on the strain’s intrinsic resistance level and the specific antibiotic involved. These results highlight that even low concentrations (0.1% *v*/*v*) of essential oils can enhance conventional antibiotic activity, albeit more reliably in moderately susceptible strains than in those with higher resistance thresholds.

Compared to previous studies, our results corroborate the antimicrobial potential of EOs. Van Vuuren et al. stated that thyme EO should be used with caution in combination with ciprofloxacin or amphotericin B, due to the predominant antagonism [18]. Mohammadzamani et al. also reported synergistic interactions between carvacrol and antibiotics against MDR *P. aeruginosa* [19]. Similarly, Vázquez-Ucha et al. demonstrated that thyme EO significantly enhanced colistin susceptibility in *K. pneumoniae* [6]. These studies support the potential use of EOs as adjuvants in antibiotic therapy to combat resistant bacterial strains.

#### 3.3.2. Assessment of the FIC Index

In order to further investigate the result, we assessed KP18 via a disk-diffusion assay and found a reduced inhibition zone (from 19 mm to 15 mm) when combining CIP with thyme EO. We also conducted a checkerboard assay. The FICI value is used to categorize the interaction of the thyme EO and ciprofloxacin. Total synergism is denoted as FICI < 0.5, additive or indifference as FICI = 0.5–4, and antagonism as FICI > 4.

The obtained FICI value of 6 (Table 5) confirms the antagonism between CIP and thyme EO in this specific clinical isolate of *K. pneumoniae*. Our finding of antagonism between ciprofloxacin and thyme essential oil (FICI = 6) in *K. pneumoniae* KP18 contrasts with earlier reports of synergistic interactions [41], where strong synergy (FICI = 0.37) between Moroccan thyme EOs and ciprofloxacin was reported. These discrepancies may stem from strain-specific resistance mechanisms, differences in essential oil chemotype or composition, and variations in experimental parameters.

#### 3.3.3. Structure-Based Antibacterial Activity of Key Antibacterial Components Is EOs

The results of various studies show that the antimicrobial effects of the EOs on the bacterial cell differ from the activities of the individual components contained in the essential oil. The antimicrobial activities of EOs are expressed due to multiple mechanisms affecting the bacterial cell targets. The effectiveness of the antimicrobial activity of aromatic compounds (terpenoids) depends significantly on their chemical structure and, consequently, their mechanism of action. The combination of the hydroxyl group and the delocalized electrons of the aromatic ring is essential for activity. The isomeric monoterpenoids (carvacrol and thymol) are strong antibacterial agents. The relative position of the hydroxyl group influences the effectiveness of the terpenes as the two isomers, thymol and carvacrol, showed different activities against Gram-positive and Gram-negative bacteria [20]. The phenol group of carvacrol is essential for modulating biophysical parameters in the cell membrane, increasing the cell membrane permeability [42]. As a result of its lipophilic character, carvacrol accumulates in the cell membrane, and as a consequence, proton exchange is disrupted. The ability of carvacrol to bind hydrogen and donate protons causes irreversible modifications in the bacterial membrane [43]. Carvacrol reduces the ATP concentration, inhibits the efflux pumps, and acts as an anti-biofilm agent by suppressing QS. The antibacterial effect of thymol against Gram-negative bacteria leads to damage to the cell membrane. This aromatic monoterpenoid causes the depolarization of cell membranes and the disturbance of intracellular homeostasis. Thymol leads to the shrinkage of bacterial cells and the destruction of the cell membrane, followed by the loss of cytoplasm, as well as DNA leakage [44]. In summary, the antibacterial activity is based on the ability of terpenoids to change the membrane-associated properties of bacteria. From this perspective, the efficiency is greater if carvacrol and thymol act synergistically with antibiotics to overcome bacterial resistance [24].

The importance of the phenolic ring was demonstrated by comparing the activity of thymol to p-cymene. The activity of p-cymene was found to be very weak [45]. Although p-cymene is weakly antimicrobial on its own, it plays an important role in the overall antimicrobial composition, and facilitates the antimicrobial properties of other substances, such as carvacrol, 4-terpineol and thymol, through synergism, antagonism and additive effects. The large accumulation of cymene in the membrane probably causes an expansion of the membrane, leading to the passive diffusion of ions between the expanded phospholipids [46].

Another element that has been shown to influence the bioactivity of essential oil components is their stereochemistry. It has been observed that α-isomers are relatively inactive compared to β-isomers (α-pinene), and cis-isomers are inactive when compared to their trans-isomers counterparts (geraniol and nerol) [45].

### 3.4. Chemical Composition by GC-MS of Thyme and Oregano EOs

As part of the investigation elucidating key antibacterial components in the studied EOs and linking their antibacterial effects to certain components, we sought to analyze the chemical compositions of the tested commercially available Bulgarian essential oils of oregano (*Origanum vulgare*) and thyme (*Thymus vulgaris*) (Table 6).

The most abundant chemical compound in the EO of *T. vulgaris* was thymol (45.74), followed by p-cymene (21.05%) and γ-terpinene (12.37%). The other compounds were present in lower concentrations, such as β-linalool (2.03%), β-caryophyllene (2.79%) and carvacrol (2.01%). The main chemical compound in the EO of *O. vulgare* was carvacrol (84.2%). Also, we detected β-linalool (3.41), p-cymene (2.82%), γ-terpinene (2.64%) and thymol (1.13%). The sum of the two phenolic monoterpenes (carvacrol and thymol) and their biosynthetic precursors p-cymene and γ-terpinene represented 90% of oregano essential oil, and 81% of thyme essential oil.

As regards other results in the literature, in her comprehensive review, Burt (2004) [22] reported that *Thymus vulgaris* essential oil contains thymol (10–64%), carvacrol (2–11%), γ-terpinene (2–31%), and p-cymene (10–56%). In contrast, *Origanum vulgare* essential oil contains carvacrol (trace-80%), thymol (trace-64%), γ-terpinene (2–52%), and p-cymene (trace-52%). Ben Selma et al. [4] reported that a GC/MS analysis revealed that *Thymus algeriensis* essential oil (TA-EO) contained 67.94% carvacrol as the major component.

Table 7 illustrates differences in chemical compositions across essential oils (EOs) derived from various studies on plant species. Ben Selma et al. (2024) reported a particularly high level of carvacrol (68%) in *Thymus algeriensis* essential oil (TA-EO), accompanied by smaller proportions of p-cymene, γ-terpinene, α-terpinyl acetate, and linalool [4]. This carvacrol-rich profile defines TA-EO as a “carvacrol chemotype”, and aligns with the strong bactericidal action against colistin-resistant *K. pneumoniae*, *P. aeruginosa*, and *Escherichia coli* reported by Ben Selma et al. [4]. The synergy between TA-EO and colistin, which reduces the antibiotic’s minimum inhibitory concentrations 16- to 512-fold and those of TA-EO by 4- to 16-fold, underscores the potential use of high-carvacrol EOs in addressing multidrug-resistant pathogens.

Comparable phenolic richness values were observed in *Thymus vulgaris* and *Origanum minutiflorum*, where thymol and carvacrol were the major compounds. For *T. vulgaris*, studies have found up to 64% thymol and 11% carvacrol, with p-cymene and γ-terpinene as common additional components [22,48]. In *O. minutiflorum*, the concentration of carvacrol was found to be 78% [39], which is among the highest reported for oregano-like plants. These phenolic compounds are frequently attributed with strong antibacterial effects [22,39], and also give rise to anti-inflammatory and antioxidative benefits in the case of *O. minutiflorum*. Another oregano species, *O. vulgare*, exhibited considerable chemotypic variation, with carvacrol ranging from trace amounts to 80% and thymol from trace to 64% [22]. Such variability may be attributed to various factors, among which we can list geographical, seasonal, and genetic factors in determining EO composition, and hence therapeutic potential. The EOs of *Melissa officinalis*, *Rosmarinus officinalis*, *Lavandula angustifolia*, and *Salvia officinalis* contained more diverse monoterpenes and other oxygenated compounds, rather than containing predominantly phenolics such as thymol or carvacrol. *M. officinalis* primarily featured citronellal, geraniol, and β-citronellol [48], whereas *R. officinalis* showed high amounts of 1,8-cineole, camphor, and α-pinene [48,49]. *L. angustifolia* was found to be rich in linalool, linalyl acetate, and, in some chemotypes, 1-dodecene [48,49], whereas *S. officinalis* contains notable quantities of α-thujone, β-thujone, 1,8-cineole, camphor, and borneol [48,49]. While these oils are often recognized for their moderate direct antibacterial action compared to carvacrol- or thymol-dominant oils, recent work illustrated how they can synergize with conventional antibiotics to enhance antibacterial efficacy against resistant microbes [49]. Clove (*S. aromaticum*), which is classically associated with high eugenol content [22], was reported to contain mainly thujone, camphor, and 1,8-cineole as its principal constituents [6]. Despite this atypical profile, synergy with colistin was still observed, mirroring the interactions reported for carvacrol-rich oils. Such synergy is relevant for tackling infections of *Acinetobacter baumannii* and *K. pneumoniae*, which are particularly resistant strains [6]. *Pogostemon cablin* (patchouli) was reported to contain significant amounts of patchouli alcohol, α-guaiene, and α-bulnesene, which contribute not only to its characteristic aroma, but also to a measurable antibacterial activity [48]. The prevalence of phenolic monoterpenes, such as carvacrol and thymol, is associated with enhanced antibacterial potential, including a reduction in antibiotic MICs against MDR pathogens. Further, the observation that combining certain EOs with antibiotics leads to heightened efficacy corroborates the potential of EOs as adjunct therapeutic tools in managing bacterial infections.

## 4. Conclusions

This investigation highlights the pressing threat of multidrug-resistant Gram-negative bacteria, particularly *P. aeruginosa* and *K. pneumoniae*, which show high prevalence in recent epidemiological data in Bulgaria and Europe. Our findings demonstrate that thyme and oregano essential oils, rich in phenolic monoterpenes like thymol and carvacrol, exert significant antibacterial and antibiofilm effects. When used alone or in combination with conventional antibiotics, these oils can disrupt bacterial membranes and enhance the overall efficacy of treatment. Consequently, thyme and oregano essential oils emerge as complementary tools to address the global challenge of AMR and reduce reliance on traditional antibiotics—particularly in regions where resistant Gram-negative infections are on the rise.

The chemical analysis of the EOs revealed high concentrations of carvacrol (84.2%) in oregano EO and thymol (45.74%) in thyme EO, which are known for their potent antibacterial properties. The minimum inhibitory concentration (MIC) and minimum bactericidal concentration (MBC) values demonstrate the superior efficacy of oregano EO compared to thyme EO against both pathogens. Furthermore, the disk diffusion assay showed that oregano EO exhibits more potent antibacterial effects than thyme EO in *K. pneumoniae* and *P. aeruginosa* strains. Even low concentrations of essential oils can enhance conventional antibiotic activity, albeit more reliably in moderately susceptible strains than in those with higher resistance thresholds.

These findings show the potential of using EOs as adjunctive therapies to enhance the efficacy of conventional antibiotics against bacterial pathogens. The use of EOs in combination with antibiotics could help to reduce the required antibiotic doses, minimize side effects, and slow down the development of antibiotic resistance. However, further research is necessary to elucidate the precise mechanisms of action, optimal concentrations, and safety profiles of these EOs in clinical settings.

## Figures and Tables

**Table 1:** Antibiotic sensitivity profile of clinical isolates.

Bacterial Strains	Clinical Isolates	Antibiotic Class			
		3GC		AG	FQ
		CTR 30	CAZ 10	GEN 10	CIP 5
*P. aeruginosa*	PA2	-	S	-	I
	PA12	-	I	-	I
	PA13	-	I	-	I
*K. pneumoniae*	KP17	R	S	S	S
	KP18	I	I	S	R

KP17—clinical isolate from sputum; KP18—clinical isolate from wound; PA2 and PA12—clinical isolates from wound; PA13—clinical isolate from sputum. Abbreviations are as follows: S, sensitive; I, intermediate; R, resistant.

**Table 2:** Antibacterial activity of oregano and thyme EO against Gram-negative strains by serial dilution method.

Antimicrobial Agent	Oregano EO Conc. % (*v*/*v*)		Thyme EO Conc. % (*v*/*v*)	
	MIC	MBC	MIC	MBC
*K. pneumoniae* ATCC13883	0.625	0.625	0.625	1.250
KP17	0.039	0.039	0.156	0.312
KP18	0.039	0.039	0.156	0.156
*P. aeruginosa* ATCC27853	1.250	1.250	2.500	5.000
PA2	0.156	0.625	2.500	2.500
PA12	0.625	2.500	5.000	5.000
PA13	0.078	0.078	1.205	1.250

Results are given as mean (n = 3). Concentrations are shown in % (*v*/*v*).

**Table 3:** Antimicrobial efficacy (inhibition zone in mm) of conventional antibiotics alone and in combination with 0.1% oregano essential oil and 0.1% thyme essential oil against *K. pneumoniae* strains.

Antimicrobial Agent/Bacterial Strains	CTR S ≥ 2 7 R < 24	CTR + Oregano EO	CTR + Thyme EO	GEN S ≥ 17 R < 17	GEN + Oregano EO	GEN + Thyme EO	CIP S ≥ 25 R < 22	CIP + Oregano EO	CIP + Thyme EO	CAZ S ≥ 22 R < 19	CAZ + Oregano EO	CAZ + Thyme EO
*K. pneumoniae* ATCC13883	35.0 ± 0.0	35.0 ± 0.0	35.0 ± 0.0	20.3 ± 0.6	30.3 ± 0.6	24.7 ± 0.6	43.7 ± 1.5	45.7 ± 0.5	44.7 ± 1.5	22.7 ± 0.6	23.7 ± 0.6	23.0 ± 0.0
KP17	22.0 ± 0.0	30.0 ± 2.0	26.0 ± 1.0	22.0 ± 0.0	22.3 ± 1.2	21.0 ± 1.7	28.7 ± 0.5	31.3 ± 1.5	28.0 ± 1.0	21.7 ± 0.6	24.7 ± 0.6	21.7 ± 0.6
KP18	24.3 ± 0.6	24.7 ± 0.6	24.3 ± 0.6	22.0 ± 0.0	22.7 ± 0.6	18.3 ± 0.6	19.3 ± 0.5	24.0 ± 1.5	15.3 ± 0.5	20.0 ± 0.0	20.0 ± 0.0	20.0 ± 0.0

CTR—ceftriaxone 30 μg; GEN—gentamicin 10 μg; CIP—ciprofloxacin 5 μg; CAZ—ceftazidime 10 μg; KP17—clinical isolate from sputum; KP18—clinical isolate from wound. EOs were used in a 0.1% concentration; experiments were conducted in triplicate.

**Table 4:** Antimicrobial efficacy (inhibition zone in mm) of conventional antibiotics alone and in combination with 0.1% oregano essential oil and 0.1% thyme essential oil against *P. aeruginosa* strains.

Antimicrobial Agent/Bacterial Strains	CIP S ≥ 50 R < 26	CIP + Oregano EO	CIP + Thyme EO	CAZ S ≥ 50 R < 17	CAZ + Oregano EO	CAZ + Thyme EO
*P. aeruginosa* ATCC27853	51.3 ± 0.6	52.7 ± 0.6	52.3 ± 0.6	55.0 ± 0.0	58.3 ± 0.6	54.7 ± 0.6
PA2	36.0 ± 2.0	55.0 ± 1.0	41.3 ± 1.2	50.7 ± 0.6	54.0 ± 1.0	49.7 ± 0.6
PA12	39.0 ± 1.0	51.7 ± 2.3	39.0 ± 1.0	22.3 ± 0.6	23.7 ± 0.6	22.3 ± 0.6
PA13	40.3 ± 1.5	41.0 ± 1.7	39.3 ± 0.6	21.0 ± 1.0	23.3 ± 1.2	21.3 ± 1.2

CIP—ciprofloxacin 5 μg; CAZ—ceftazidime 10 μg; PA2 and PA12—clinical isolates from wound; PA13—clinical isolate from sputum. EOs were used in 0.1% concentration; experiments were conducted in triplicate.

**Table 5:** Checkerboard assay of KP18.

Strain	MICa (Thyme EO)	A	FICA	MICb (CIP)	B	FICB	FICI
KP18	0.156	0.625	4	8	16	2	6

**Table 6:** Chemical compositions of oregano and thyme essential oil.

Name	Oregano	Thyme	RT	RI
α-Thujene	nd	1.34	9.11	922
α-Pinene	0.32	1.26	9.32	929
Camphene	0.13	1.11	9.84	945
β-Pinene	nd	0.15	10.75	974
1-Octen-3-ol	nd	0.2	10.97	980
β-Myrcene	0.69	0.88	11.23	988
α-Phellandrene	0.12	nd	11.71	1004
α-Terpinene	1.03	1.13	12.06	1015
p-Cymene	2.82	21.05	12.31	1023
Limonene	nd	0.5	12.47	1027
γ-Terpinene	2.64	12.37	13.41	1057
Sabinene hydrate	nd	0.57	13.79	1069
β-Linalool	3.41	2.03	14.74	1099
Camphor	nd	1.01	16.12	1145
Borneol	0.48	1.79	16.9	1171
Terpinen-4-ol	nd	0.87	17.16	1180
α-Terpineol	0.48	nd	17.63	1195
Thymol methyl ether	nd	0.14	18.58	1228
Carvacrol, methyl ether	nd	1.33	18.84	1237
Bornyl acetate	nd	0.66	20.2	1286
Thymol	1.13	45.74	20.37	1293
Carvacrol	84.2	2.01	20.75	1301
β-Caryophyllene	0.74	2.79	23.77	1420
Aromadendrene	0.09	nd	24.23	1438
β-Bisabolene	1.13	nd	25.93	1506
γ-Cadinene	nd	0.12	26.07	1512
δ-Cadinene	nd	0.18	26.19	1517
Caryophyllene oxide	0.11	0.48	27.73	1581

Cells represent % of total TIC; RI—retention index from the literature [47]; RT—retention time; nd—not detected.

**Table 7:** Chemical profiles of different EOs.

Essential Oil	Dominant Compound(s)	Reference
*Thymus algeriensis*	Carvacrol (68%), p-Cymene (9%), γ-Terpinene (3%), α-Terpinyl acetate (2%), Linalool (1%)	[4]
*Thymus vulgaris*	Thymol (10–64%), Carvacrol (2–11%), γ-Terpinene (2–31%), *p*-Cymene (10–56%)	[22]
	Thymol (52%), p-Cymene (18%), Carvacrol (4%), 1,8-Cineole (6%), α-Terpineol (3%)	[48]
*Origanum minutiflorum*	Carvacrol (78%), Linalool (5%), *p*-Cymene (4%), γ-Terpinene (2%), (E)-Caryophyllene (2%)	[39]
*Origanum vulgare*	Carvacrol (Trace-80%), Thymol (Trace-64%), γ-Terpinene (2–52%), *p*-Cymene (Trace-52%)	[22]
*Melissa officinalis*	Citronellal (21%), Geraniol (17%), β-Citronellol (12%), Caryophyllene Oxide (1%), α-Cadinol (3%)	[48]
*Rosmarinus officinalis*	1,8-Cineole (48%), Camphor (10%), α-Pinene (10%), Borneol (8%)	[49]
	1,8-Cineole (29%), Camphor (17%), α-Pinene (12%), β-Pinene (6%), Limonene (5%)	[48]
*Lavandula angustifolia*	Linalool (30%), 1-Dodecene (33%)	[49]
	Linalool (25%), Linalyl Acetate (23%), 1,8-Cineole (3%), Terpinen-4-ol (5%), β-Farnesene (3%)	[48]
*Salvia officinalis*	Thujone isomers (25%), Camphor (22%)	[49]
	α-Thujone (16%), β-Thujone (11%), 1,8-Cineole (27%), Borneol (10%), Camphor (8%)	[48]
*Syzygium aromaticum*	Thujone (25%), Camphor (22%), 1,8-Cineole (Eucalyptol) (11%), Thujone-Trans (7%), Caryophyllene (4%)	[6]
*Pogostemon cablin*	Patchouli Alcohol (23%), α-Guaiene (14%), γ-Patchoulene (9%), β-Patchoulene (7%), α-Bulnesene (17%)	[48]

## Data Availability

The dataset used and analyzed during the current study is available from the corresponding author on request.

## Abbreviations

The following abbreviations are used in this manuscript:

3GC	Third-generation cephalosporins
AG	Aminoglycosides
AMR	Antimicrobial resistance
ATCC	American Type Culture Collection
BPPL	Bacterial Priority Pathogens List
CARB	Carbapenems
CAUTI	Catheter-associated urinary tract infection
CAZ	Ceftazidime
CDC	Centers for Disease Control and Prevention
CFU	Colony-forming units
CIP	Ciprofloxacin
CLABSI	Central line-associated bloodstream infection
COVID-19	Coronavirus disease 2019
CR3	Combined resistance to three or more antibiotic groups (among PIT, CAZ, CARB, FQ, and AG)
CRKP	Carbapenem-resistant *K. pneumoniae*
CRPA	Carbapenem-resistant *P. aeruginosa*
CTR	Ceftriaxone
DMSO	Dimethyl sulfoxide
EARS-Net	European Antimicrobial Resistance Surveillance Network
ECDC	European Centre for Disease Prevention and Control
EMA	European Medicines Agency
EO	Essential oil
EU/EEA	European Union/European Economic Area
EUCAST	European Committee on Antimicrobial Susceptibility Testing
FQ	Fluoroquinolones
GC-MS	Gas chromatography-mass spectrometry
GEN	Gentamicin
GDP	Gross domestic product
HAI	Healthcare-associated infection
ICU	Intensive care unit
IFRA	International Fragrance Association
*K. pneumoniae*	*Klebsiella pneumoniae*
MBC	Minimum bactericidal concentration
MDR	Multidrug-resistant
MIC	Minimum inhibitory concentration
MSD	Mass selective detector
NIST	National Institute of Standards and Technology
*P. aeruginosa*	*Pseudomonas aeruginosa*
PDR	Pandrug-resistant
PIT	Piperacillin–tazobactam
QS	Quorum sensing
RI	Retention index
RT	Retention time
SSI	Surgical site infection
TA-EO	*Thymus algeriensis* essential oil
TIC	Total ion current
WHO	World Health Organization
XDR	Extensively drug-resistant
